# Impact of BMI on Organs at Risk Dose for Cervical Cancer Treated With Definitive Radiation Therapy Followed by Brachytherapy Boost

**DOI:** 10.7759/cureus.104301

**Published:** 2026-02-26

**Authors:** Justin Tang, Caroline Oska, Xianhong Xie, Ilan Small, Keyur J Mehta, Ryung S Kim, Ravindra Yaparpalvi

**Affiliations:** 1 Radiation Oncology, Montefiore Medical Center/Albert Einstein College of Medicine, Bronx, USA; 2 Epidemiology and Public Health, Albert Einstein College of Medicine, Bronx, USA; 3 Radiation Oncology, Albert Einstein College of Medicine, Bronx, USA

**Keywords:** bmi, brachytherapy, cervix, hdr ir-192, hybrid applicator

## Abstract

Objective

Higher body mass index (BMI) is identified as a protective factor in intracavitary brachytherapy (BT) for cervical cancer (CC) treated with definitive radiation therapy, particularly for gastrointestinal toxicity. The impact of BMI on organs at risk (OAR) dose remains unknown as hybrid intracavitary-interstitial applicators are used.

Methods

Patients with CC who received definitive chemoradiation followed by BT boosts from 2023 to 2025 were included. BT doses to OAR of 0.1cm^3^, 1cm^3^, and 2cm^3^ were collected as % of the prescription dose (%Rx). A linear mixed effects regression model was fitted to evaluate the relationship between BMI and dose to OARs.

Results

Fifty-six patients with 221 BT plans were included. The majority of patients included were 2018 International Federation of Gynecology and Obstetrics (FIGO) stage IIIC (n = 29, 51.8%). The mean ± standard deviation (SD) of BMI for the cohort was 29.4 ± 7.6 kg/m^2^, with 36 (64.3%) patients receiving hybrid BT with a median (interquartile range (IQR)) of 2 (2-3) needles. The mean ± SD of D0.1cm^3^, D1cm^3^, and D2cm^3^ %Rx for rectum were 55.4% ± 18.6, 45% ± 15.4, and 41.4% ± 13.9; for sigmoid were 63.9% ± 17.2, 52.5% ± 14.3, and 47.8% ± 13.3; for bladder were 83.3% ± 13.6, 71.0% ± 11.2, and 65.6% ± 10.6; and for small bowel (SB) were 51.4% ± 22.9, 42.1% ± 19.6, and 38.0% ± 17.5, respectively. While controlling for other variables, each one-point increase in BMI decreased the small bowel D0.1cm^3^, D1cm^3^, and D2cm^3^ by 0.91, 0.78, and 0.72, respectively (p = 0.008 for D0.1cm^3^, and 0.006 for D1cm^3 ^and D2cm^3^). The bladder D1cm^3^ and D2cm^3^ decreased by 0.31 (p=0.030) and 0.32 (p=0.017), respectively. The same was not true for the rectum and sigmoid.

Conclusion

We found an inverse relationship between BMI and dose to the small bowel and bladder during BT.

## Introduction

The standard treatment for locally advanced cervical cancer (LACC) is external beam radiotherapy (EBRT) and concurrent chemotherapy followed by brachytherapy (BT) boost with or without immunotherapy [1]. Image-guided brachytherapy (IGBT) is a modern technique that achieves excellent outcomes for LACC while sparing organs at risk (OAR) in several studies [2-5]. However, while advancements in BT limit severe treatment toxicities, mild morbidities and their impact on patient quality of life remain prevalent [6].

EMBRACE-1 was a prospective, observational, cohort study evaluating local control (LC) and late morbidity for patients with LACC treated with chemoradiotherapy followed by MRI-based IGBT [7]. They achieved an actuarial five-year LC rate of 92% (similar across all International Federation of Gynecology and Obstetrics (FIGO) stages) and rates of grade 3-5 genitourinary, gastrointestinal (GI), and vaginal morbidity of 6.8%, 8.5%, and 5.7%, respectively [7]. RetroEMBRACE similarly evaluated LC and toxicity among patients treated with IGBT for LACC [5]. While achieving an excellent five-year LC of 89%, their rates of grade 3-5 morbidity for the bladder, GI system, and vagina were 5%, 7%, and 5%, respectively [5]. In both studies, GI toxicity, while still <10% for grade 3-5, was the greatest contributor to patient toxicity. A subsequent study evaluating women on EMBRACE found that while severe bowel toxicity rates were low, rates of grade 1-2 bowel morbidity affected up to 1/3 of patients and caused a substantial burden to the quality of their lives [6].

Due to the high prevalence and burden of GI toxicity in patients with LACC, efforts have been made to identify factors that place patients at increased risk, such as increased dose to various segments of the GI tract [8]. However, the likely contribution is multifactorial as doses to various OAR can cause bile acid malabsorption, small bowel (SB) bacterial overgrowth, rapid bowel transit, abnormal fiber intake, or stricture formation, with components of each adding together to ultimately cause GI symptoms such as fecal incontinence [8]. One study with this aim found that while treatment-related factors, such as rectum D2cm^3^, contribute to persistent GI toxicity, certain patient-specific factors also play a role [8]. For example, those who were underweight in EMBRACE-I were found to be at a higher risk for proctitis and rectal bleeding [8]. Another study evaluating patients with LACC found that those with a body mass index (BMI) < 18.5 kg/m^2^ were not only found to have a greater risk of grade 3-4 treatment-related complications compared to those with BMI > 24.9 kg/m^2^, but they also had significantly decreased overall survival (p < 0.01) [9].

While a few additional studies have since evaluated the impact of BMI on toxicity in gynecologic malignancies, the BT dose contribution is not well studied in the setting of hybrid intracavitary/interstitial BT applicators [9-11]. Given the possible prognostic implication of BMI, we sought to characterize its effect in a cohort predominantly treated with hybrid applicators, a BT technique that specifically intends to improve target coverage while better sparing OAR dose. We hypothesized that higher BMI would decrease the small bowel (SB) dose delivered in high-dose-rate (HDR) BT.

## Materials and methods

This was a single-center, retrospective, observational study done at an urban academic medical center in the Bronx, NY. Patients were included if they received definitive chemoradiation followed by an IGBT boost for CC from 2023 to 2025. Baseline patient characteristics, including age, sex, BMI, and initial stage, were collected at the time of their BT boosts. BT doses to 0.1cm^3^, 1cm^3^, and 2cm^3^ were collected as percentage of prescription dose (%Rx) for our included OAR (rectum, sigmoid, SB, and bladder). All dose and optimization calculations were performed using the American Association of Physicists in Medicine (AAPM) TG-43 dose calculation formalism within a commercial high-dose-rate (HDR) brachytherapy treatment planning system (Oncentra version 4.6; Elekta AB, Stockholm, Sweden). Treatments were delivered using a Flexitron HDR afterloader (Elekta, Stockholm, Sweden). Contours of target and OAR volumes were completed following ABS guidelines; small bowels were contoured as bowel loops near the target structures.

For descriptive statistics, mean (standard deviation (SD)) and median (interquartile range (IQR)) on the continuous variables, and number (%) on the categorical variables were calculated. For each OAR and each dose volume histogram (DVH) parameter (D0.1cm^3^, D1cm^3^, and D2cm^3^), a linear mixed effects regression model was fitted with the radiation dose as the outcome, BMI as the predictor of interest, age and log(high-risk clinical target volume (HR-CTV)) as the potential confounders, and subject effects as random intercepts to account for correlation among the repeated measurements from same patients. Sensitivity analyses were performed for these linear mixed effects models by removing a patient with the three largest BMIs (leverage/influential points). In addition, a similar linear mixed effect model was fitted with the log(HR-CTV) as the outcome, BMI as the predictor of interest, and the subject effects as random intercepts, with and without adjusting for age as a potential confounder. SAS software version 9.4 (SAS Inc., Cary, NC) was used for statistical analyses.

## Results

Between 2023 and 2025, 56 patients with CC completed definitive chemoradiation, which included a BT boost. Baseline patient characteristics are summarized in Table 1. The median (IQR) age at the time of BT was 54 (42-62) years. The mean ± SD BMI was 29.4 ± 7.6 kg/m^2^. The most common pre-treatment 2018 FIGO stage in our population was IIIC1 (n = 22, 39.3%), followed by IIIC2 (n = 7, 12.5%) and IIB (n = 7, 12.5%). Regarding the American Joint Committee on Cancer (AJCC) ninth edition T staging, 18 (32.1%) patients had parametrial involvement (T2B), and another nine (16.1%) had pelvic sidewall involvement (T3B) at diagnosis. Over half of the patients had lymph node involvement at diagnosis (n = 29, 51.8%). One patient with FIGO IA was treated definitively with chemoradiation followed by brachytherapy boost due to simultaneous diagnosis of locally advanced vulvar cancer.

**Table 1:** Characteristics of the patients at baseline † M1 patients had inguinal nodal involvement and were treated definitively (AJCC ninth edition). AJCC: American Joint Committee on Cancer, BMI: body mass index, FIGO: International Federation of Gynecology and Obstetrics, IQR: interquartile range, SD: standard deviation

Characteristics	Total (N = 56)
Age, year	
Mean ± SD	53.4 ± 15.2
BMI	
Median (IQR)	27.8 (26.1-32.6)
2018 FIGO stage, number (%)	
IA	1 (1.8)
IB	6 (10.7)
IIA	8 (14.3)
IIB	7 (12.5)
IIIA	1 (1.8)
IIIB	2 (3.6)
IIIC1	22 (39.3)
IIIC2	7 (12.5)
IVB	2 (3.6)
AJCC T stage, number (%)	
T1a	2 (3.6)
T1b	14 (25.0)
T2a	12 (21.4)
T2b	18 (32.1)
T3a	1 (1.8)
T3b	9 (16.1)
AJCC N stage, number (%)	
N0	27 (48.2)
N1	22 (39.3)
N2	7 (12.5)
AJCC M stage, number (%)	
M0	54 (96.4)
† M1	2 (3.6)

Regarding treatment characteristics (Table 2), 36 (64.3%) patients were treated with hybrid BT applicators, while the remaining 20 (35.7%) were treated solely with standard intracavitary devices, such as tandem and ovoids. The median (IQR) number of needles used per fraction was 2 (2-3), and the most common fractionation regimen used was 7 Gy × 4 fractions, with 49 (87.5%) patients receiving this regimen.

**Table 2:** Brachytherapy treatment characteristics HR-CTV: high-risk clinical target volume, IQR: interquartile range, SD: standard deviation

Treatment characteristics	Total (N = 56)
Applicator type, number (%)	
Intracavitary	20 (35.7)
Intracavitary-interstitial	36 (64.3)
Interstitial needles, median (IQR)	2 (2-3)
Number of fractions, median (IQR)	4 (4-4)
Dose (Gy), number (%)	
6	3 (5.4)
7	49 (87.5)
8	4 (7.1)
HR-CTV (cc)	
Mean ± SD	26.0 ± 14.0
Rectal %Rx dose, mean ± SD	
D_0.1cm_^3^	55.4 ± 18.6
D_1cm_^3^	45.5 ± 15.4
D_2cm_^3^	41.4 ± 13.9
Sigmoid %Rx dose, mean ± SD	
D_0.1cm_^3^	63.9 ± 17.2
D_1cm_^3^	52.5 ± 14.3
D_2cm_^3^	47.8 ± 13.3
Bladder %Rx dose, mean ± SD	
D_0.1cm_^3^	83.3 ± 13.6
D_1cm_^3^	71.0 ± 11.2
D_2cm_^3^	65.6 ± 10.6
Small bowel %Rx dose, mean ± SD	
D_0.1cm_^3^	51.4 ± 22.9
D_1cm_^3^	42.1 ± 19.6
D_2cm_^3^	38.0 ± 17.5

BT plan evaluation revealed that the mean ± SD HR-CTV was 26.0 ± 14.0 cc. The mean D0.1cm^3^, D1cm^3^, and D2cm^3^ %Rx ± SD for rectum were 55.4 ± 18.6, 45 ± 15.4, and 41.4 ± 13.9; for sigmoid were 63.9 ± 17.2, 52.5 ± 14.3, and 47.8 ± 13.3; for bladder were 83.3 ± 13.6, 71.0 ± 11.2, and 65.6 ± 10.6; and for SB were 51.4 ± 22.9, 42.1 ± 19.6, and 38.0 ± 17.5, respectively.

A mixed effects model analysis for the association between BMI and log(HR-CTV) revealed no significant relationship between the two. However, for log(HR-CTV) and SB dose, there was a significant association for D0.1cm^3^ and D2cm^3^. As expected, there is a direct association between HR-CTV and dose to the small bowel. The log2(HR-CTV) coefficients (95% CI) were 5.03 (0.14, 9.92), p=0.044, and 4.10 (0.49, 7.71), p=0.026, respectively, which can be interpreted as the effects of doubling of HR-CTV on the increases of small bowel radiation doses D0.1cm^3^ and D2cm^3^, controlling for BMI and age.

**Figure 1:**
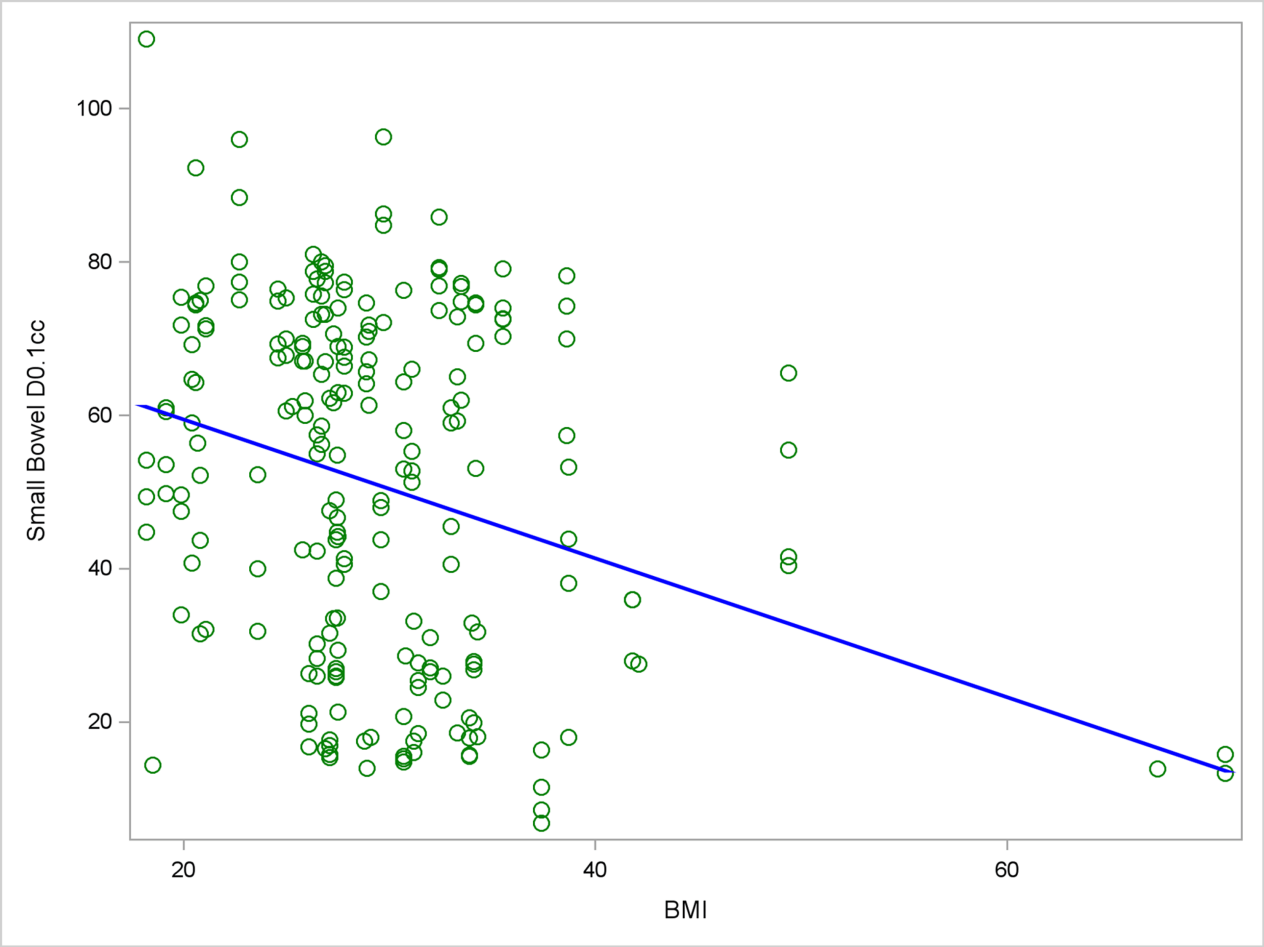
Scatterplot of small bowel D0.1cc versus BMI overlaid with the linear mixed effect model fitted line Each circle in the plot represents a patient/fraction. The linear mixed effect model with the outcome D0.1cc, the predictor BMI, the potential confounders (age and log-transformed HR-CTV), and the random subject effect was being fitted. The slope of BMI on small bowel D0.1cc is -0.91 (95% CI: -1.57, -0.24; p = 0.008). The covariates age and log-transformed HR-CTV are set to their mean values, respectively, in drawing the fitted line. BMI: body mass index, HR-CTV: high-risk clinical target volume

Mixed effect models for dose to OAR and BMI were subsequently performed while controlling for the log(HR-CTV) and age (Table 3). This analysis revealed a significantly inverse correlation between BMI and dose to SB. While controlling for age and log(HR-CTV), each one-point increase in BMI decreased the SB D0.1cm^3^ by 0.91 with 95% CI of -1.57 to -0.24 (p = 0.008). The SB D1cm^3^ and D2cm^3^ also decreased by 0.78 with 95% CI of -1.34 to -0.22 (p = 0.006) and 0.72 with 95% CI of -1.23 to -0.18 (p = 0.006), respectively. The finding for SB D0.1cm^3^ is depicted in Figure 1.

**Table 3:** Impact of BMI on OAR dose using mixed effects models Mixed effects models adjusting for age and log(HR-CTV) were used. *p<0.05 with an inverse relationship between BMI and OAR dose BMI: body mass index, CI: confidence interval, HR-CTV: high-risk clinical target volume, OAR: organs at risk

Organ	Parameter	Change of OAR dose per 1 BMI increase (95% CI)	P-value
Rectum	D_0.1cm_^3^	0.50 (0.04, 0.97)	0.035
Rectum	D_1cm_^3^	0.46 (0.08, 0.85)	0.019
Rectum	D_2cm_^3^	0.43 (0.08, 0.78)	0.016
Sigmoid	D_0.1cm_^3^	-0.03 (-0.45, 0.39)	0.892
Sigmoid	D_1cm_^3^	-0.003 (-0.36, 0.35)	0.988
Sigmoid	D_2cm_^3^	-0.03 (-0.36, 0.30)	0.845
Bladder	D_0.1cm_^3^	-0.29 (-0.64, 0.05)	0.094
Bladder	D_1cm_^3^	-0.31 (-0.59, -0.03)	0.030*
Bladder	D_2cm_^3^	-0.32 (-0.59, -0.06)	0.017*
Small bowel	D_0.1cm_^3^	-0.91 (-1.57, -0.24)	0.008*
Small bowel	D_1cm_^3^	-0.78 (-1.34, -0.22)	0.006*
Small bowel	D_2cm_^3^	-0.72 (-1.23, -0.21)	0.006*

The bladder also demonstrated an inverse relationship between dose and BMI, such that each one-point increase in BMI decreased the D1cm^3^ by 0.31 with 95% CI of -0.59 to -0.03 (p = 0.030) and the D2cm^3^ by 0.32 with 95% CI of -0.59 to -0.06 (p = 0.017). Contrastingly, the dose to the rectum actually demonstrated a direct correlation with BMI, while there was no significant association between BMI and dose to the sigmoid colon.

In our cohort, there was one patient with a significantly elevated BMI of 70.6, who contributed three data points due to having received three fractions of BT. To determine whether our results remained true without this outlier, we performed a sensitivity analysis excluding this patient’s three data points. For SB, the D0.1cm^3^ remained significant; each one-point increase in BMI decreased the D0.1cm^3^ by 0.81 with 95% CI of -1.60 to -0.03 (p = 0.041). The SB D1cm^3^ lost significance, although there remained a trend toward an inverse correlation, as the SB D1cm^3^ decreases by 0.70 for each one-point BMI increase with a 95% CI of -1.48 to 0.09 (p=0.079). Similarly, for the bladder, only D0.1cm^3^ showed significance with each one-point increase in BMI, decreasing the D0.1cm^3^ by 0.45 with 95% CI of -0.84 to -0.06 (p=0.025). The bladder’s D1cm^3^ was trending toward significance with a decrease of 0.39 and 95% CI of -0.78 to 0.00 (p=0.053). Regarding the other OARs, the rectum dose lost its significant direct correlation with a BMI increase, and the relationship to sigmoid colon dose remained nonsignificant.

Subsequently, we performed a separate analysis stratifying the patients by intracavitary versus hybrid applicator (Appendices). The inverse relationship of BMI and small bowel remains statistically significant for hybrid only, but not for intracavitary applicator. However, there was a statistically significant direct relationship between BMI and rectal dose for the hybrid applicator as well when we performed the stratification. 

## Discussion

In our cohort of patients treated with HDR BT for CC between 2023 and 2025, we found an association between BMI and SB dose, such that those with higher BMI received a significantly lower dose to their SB after controlling for age and target volume. This was true and significant for all SB D0.1cm^3^, D1cm^3^, and D2cm^3^. The bladder was another OAR with a significantly inverse correlation between dose and BMI, although only for D1cm^3^ and D2cm^3^. After performing a sensitivity analysis and eliminating the outlier with a BMI of 70.6, the results held true for SB D0.1cm^3^ and trended toward significance for D1cm^3^. Similarly, there was a trend toward a significantly inverse correlation between BMI and D1cm^3^ for bladder, although only D0.1cm^3^ was statistically significant. In our study, there was no inverse relationship between BMI and dose to the rectum or sigmoid colon. The placement of interstitial needles may have negated some of the buffering effect of fatty tissues between the target and the rectum.

In agreement with the EMBRACE-I findings, a study by Lim et al. assessed rectal doses in 51 patients with LACC treated with definitive EBRT and HDR BT [10]. They found a significant decrease in the D1cm^3^ and D2cm^3^ rectal dose (p = 0.016), ICRU rectal point dose (p = 0.022), and mean rectal dose percentage (p = 0.021) with an increase in BMI [10]. In this study, the dose was normalized to point A, SB doses were unassessed, and only intracavitary BT devices were used. The discrepant relationship between BMI and rectal dose in our study and these others indicates that further research on this relationship using hybrid intracavitary-interstitial BT, rather than just intracavitary, is warranted. Regarding the SB dose in EMBRACE-I, the SB D2cm^3^ correlated with grade 3 or more sigmoid and small bowel events, as well as with moderate/persistent diarrhea and flatulence [8]. Importantly, these were two of the most frequently reported late GI effects, and they lasted through over two years of median follow-up [6,8]. When we stratified our cohort by hybrid versus intracavitary applicator, the inverse relationship between BMI and small bowel was only seen in the hybrid group. This observation is likely due to the placement of interstitial needles for dose optimization to reduce dose to the small bowel while dumping dose to nearby adipose tissue, leading to a statistically significant slight increase in rectal dose.

Our outcomes are relevant to other previously published literature evaluating the impact of BMI on patients with gynecologic malignancies treated with radiotherapy. One study by Lee et al. evaluated the effects of BMI and weight change during radiotherapy on the development of toxicity for patients with LACC [11]. After a 63-month median follow-up, the five-year rates of grade 3 or higher late GI toxicities were 18.6%, 4.0%, and 4.2% for the underweight, normal weight, and overweight groups, respectively (p = 0.002) [11]. Weight loss during treatment was also a significant predictor for the development of grade 3 or higher late GI toxicity (p = 0.004) [11]. Patients in this study were treated only with intracavitary BT boosts, which is in contrast to our study. However, another study found more staggering findings, such that BMI appeared to not only impact toxicity but also survival outcomes in patients with LACC. This previously mentioned study by Kizer et al. found five-year overall survival rates of 33%, 60%, and 68% for a BMI of <18.5 kg/m^2^, 18.5-24.9 kg/m^2^, and >24.9 kg/m2, respectively [9]. In other words, underweight patients have roughly half the overall survival of both normal weight patients and those who are overweight or obese. While this may be partly attributed to confounding baseline factors, such as more underweight patients being smokers and having higher stages of disease, they also suffered a significantly increased risk for grade 3-4 complications from radiotherapy [9]. These included radiation enteritis (p = 0.03), fistula (p = 0.05), and bowel obstruction (p < 0.001), which may all contribute to fatality [9].

These findings provide a unique example of higher BMI serving as a protective factor in treatment toxicities. It is well known that obesity increases the risk of developing various cancers in the United States, including liver, endometrial, breast, colon, and rectal, among others [12]. Moreover, the incidence of various cancers is rising among younger adults, and this is attributed to a rise in body weight in at least four types (colon, rectal, pancreatic, and kidney) [13]. Even after successful treatment, obesity is detrimental to several aspects of cancer survivorship, including cancer recurrence, risk of developing secondary cancers, and quality of life [14-17]. Perhaps more relevant to the present study, patients with increasing BMI are more likely to be diagnosed with invasive cancer versus pre-cancerous lesions on cervical cancer screening [18]. One study evaluating 944,227 women who underwent cytology and human papillomavirus (HPV) testing found that obese women had the lowest five-year risk of precancer (p < 0.001) and the highest five-year risk of cancer (p < 0.001) compared to their lesser weight counterparts [18]. This was attributed to difficulties with visualization and sampling, although many other biology-based mechanisms are implicated in causing worse oncological outcomes in obese patients. These include chronic inflammation, excess estrogen, and impairments of cellular autophagy [19,20]. While the best way to prevent toxicities is through modifications of risk factors for cancerization, our findings and those of others suggest that the silver lining for these patients is that higher BMI may render the treatment less toxic on surrounding OARs once they are diagnosed with cancer. These findings may be important in the context of the increased prevalence of weight loss medications such as GLP-1 agonists and whether these medications should be paused while on treatment.

The mechanism for higher BMI serving as a protective factor for patients with LACC undergoing radiotherapy is less established. The most plausible explanation is that more mesorectal fat better attenuates the dose received by OARs during EBRT and BT boosts. With visceral fat providing a greater distance between the BT source and OARs, a sparing effect can be expected due to the inverse square law. Underweight patients do not have this cushion and thus experience higher OAR doses and, therefore, greater toxicity. A similar sparing effect is seen in patients with higher BMIs who receive prostate BT with permanent I-125 sources [21]. In our study, this effect was most apparent when evaluating the SB dose, which is a major cause of lasting morbidity among survivors of LACC.

Our study offered a dosimetric analysis of the relationship between OAR and BMI. The strength of this study is the individual dosimetric data and the higher BMI of the cohort, which is more relevant to the US population. The limitation of our studies is the small sample size and the lack of toxicity data to correlate with the D2cm^3^ findings. However, D2cm^3^ correlations to toxicities are well published [3]. Another limitation is that BMI cannot distinguish between visceral and subcutaneous fat distribution, of which visceral fat would be of more assistance dosimetrically for brachytherapy to push OAR away from the treatment target. Along the same line, BMI does not account for changes in muscle mass that occur with aging. Our models attempted to correct that by including age as a confounder. In addition, we separately calculated body fat percentage, and the inverse relationship is still maintained.

## Conclusions

Our study demonstrated that there is an inverse dosimetric relationship between small bowel and BMI, possibly due to the distance created by visceral fat. However, patients with lower BMI are likely to have less visceral fat, leading to a higher dose to OAR. One can conclude from available literature, including the present study, that patients with CC with lower BMIs at diagnosis may warrant special attention to limit and/or alleviate GI toxicity from treatment. Weight can therefore serve as a modifiable risk factor that treating radiation oncologists can identify and act on. Various resources exist to address this issue, including more diligent weight tracking, more frequent on-treatment visits for monitoring, prescriptions for nutritional supplements, and referrals to dieticians. Knowledge of treatment-related BT parameters affected by BMI, in addition to their known effects on lasting toxicity and prognostication, justifies this extra diligence and attention.

## Appendices

Table 4 presents the impact of BMI on OAR dose with patients stratified by intracavitary versus hybrid applicator.

**Table 4:** Impact of BMI on OAR dose using mixed effects models stratified by applicator type (intracavitary or hybrid) Mixed effects models adjusted for age and log(HR-CTV) stratified by applicator type were used. *p < 0.05 with an inverse relationship between BMI and OAR dose BMI: body mass index, CI: confidence interval, HR-CTV: high-risk clinical target volume, OAR: organs at risk

Organ	Area	Intracavitary		Hybrid		P for BMI by applicator type interaction
		BMI coefficient (95% CI), change of OAR dose per 1 BMI increase (95% CI)	P-value	BMI coefficient (95% CI), change of OAR dose per 1 BMI increase (95% CI)	P-value	
Rectum	D0.1cc	0.13 (-0.47, 0.73)	0.67	0.81 (0.11, 1.51)	0.02*	0.14
Rectum	D1cc	0.19 (-0.31, 0.68)	0.46	0.70 (0.12, 1.28)	0.02*	0.18
Rectum	D2cc	0.19 (-0.26, 0.65)	0.40	0.64 (0.11, 1.17)	0.02*	0.20
Sigmoid	D0.1cc	-0.30 (-0.85, 0.25)	0.29	0.10 (-0.53, 0.73)	0.75	0.35
Sigmoid	D1cc	-0.24 (-0.71, 0.22)	0.30	0.15 (-0.38, 0.69)	0.58	0.27
Sigmoid	D2cc	-0.26 (-0.69, 0.17)	0.24	0.12 (-0.38, 0.62)	0.64	0.25
Bladder	D0.1cc	-0.31 (-0.74, 0.11)	0.15	-0.18 (-0.67, 0.32)	0.48	0.68
Bladder	D1cc	-0.37 (-0.71, -0.02)	0.04*	-0.15 (-0.55, 0.25)	0.46	0.42
Bladder	D2cc	-0.39 (-0.72, -0.06)	0.02*	-0.15 (-0.53, 0.23)	0.45	0.33
Small bowel	D0.1cc	-0.65 (-1.56, 0.25)	0.16	-1.54 (-2.60, -0.47)	0.005*	0.19
Small bowel	D1cc	-0.57 (-1.31, 0.16)	0.13	-1.19 (-2.06, -0.33)	0.01*	0.26
Small bowel	D2cc	-0.53 (-1.22, 0.15)	0.12	-1.17 (-1.98, -0.37)	0.004*	0.21
